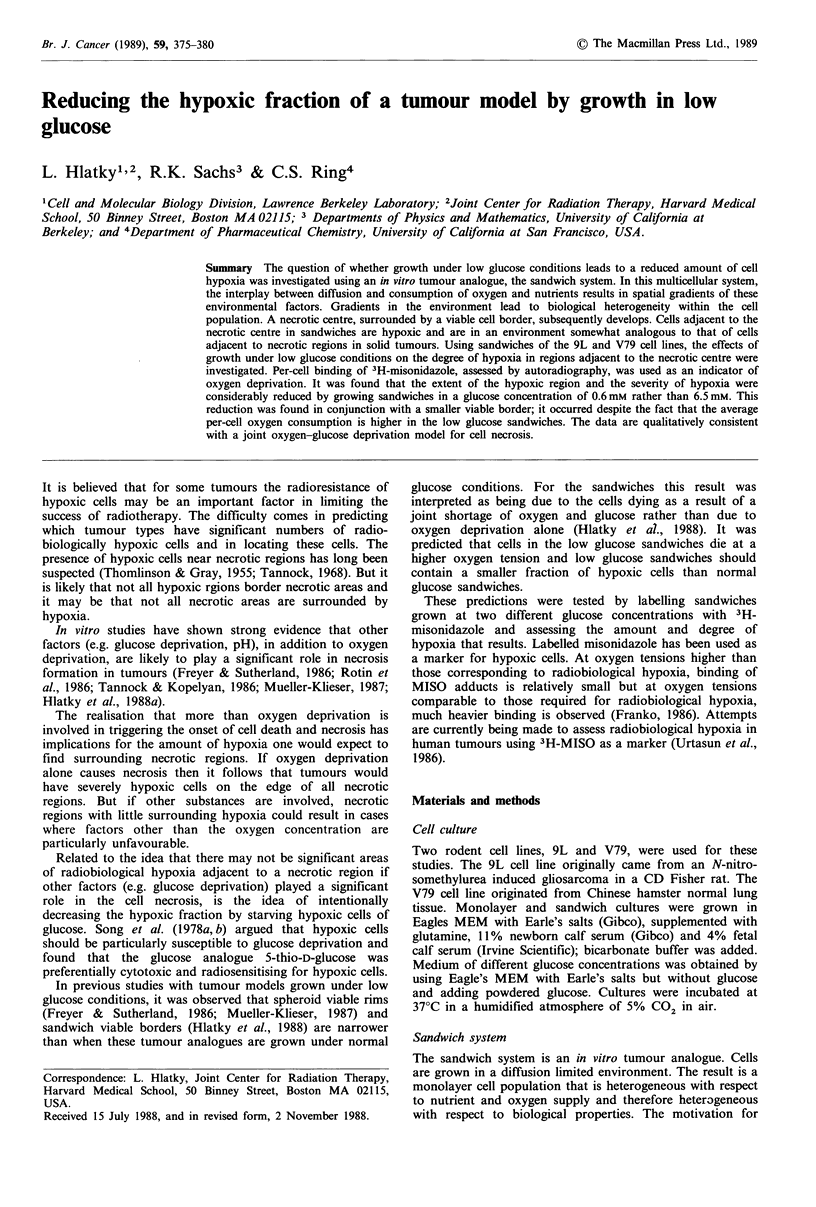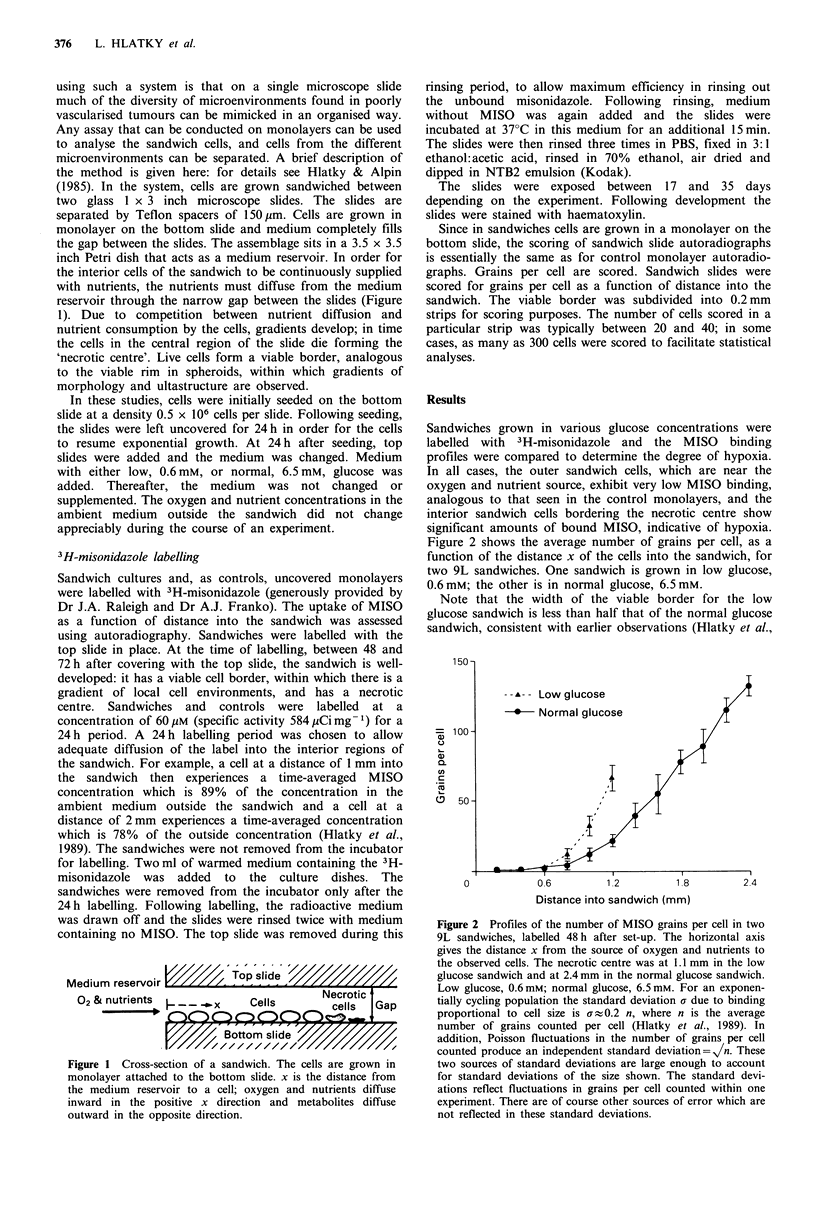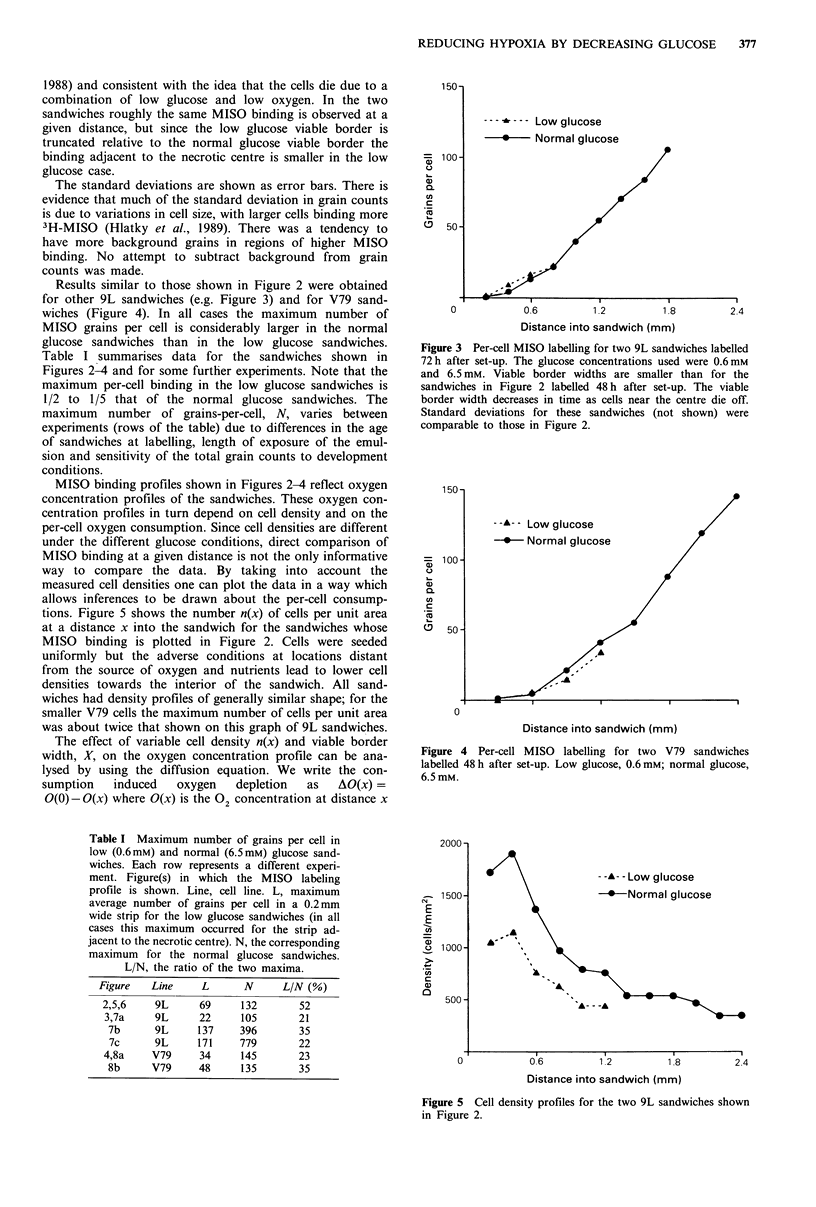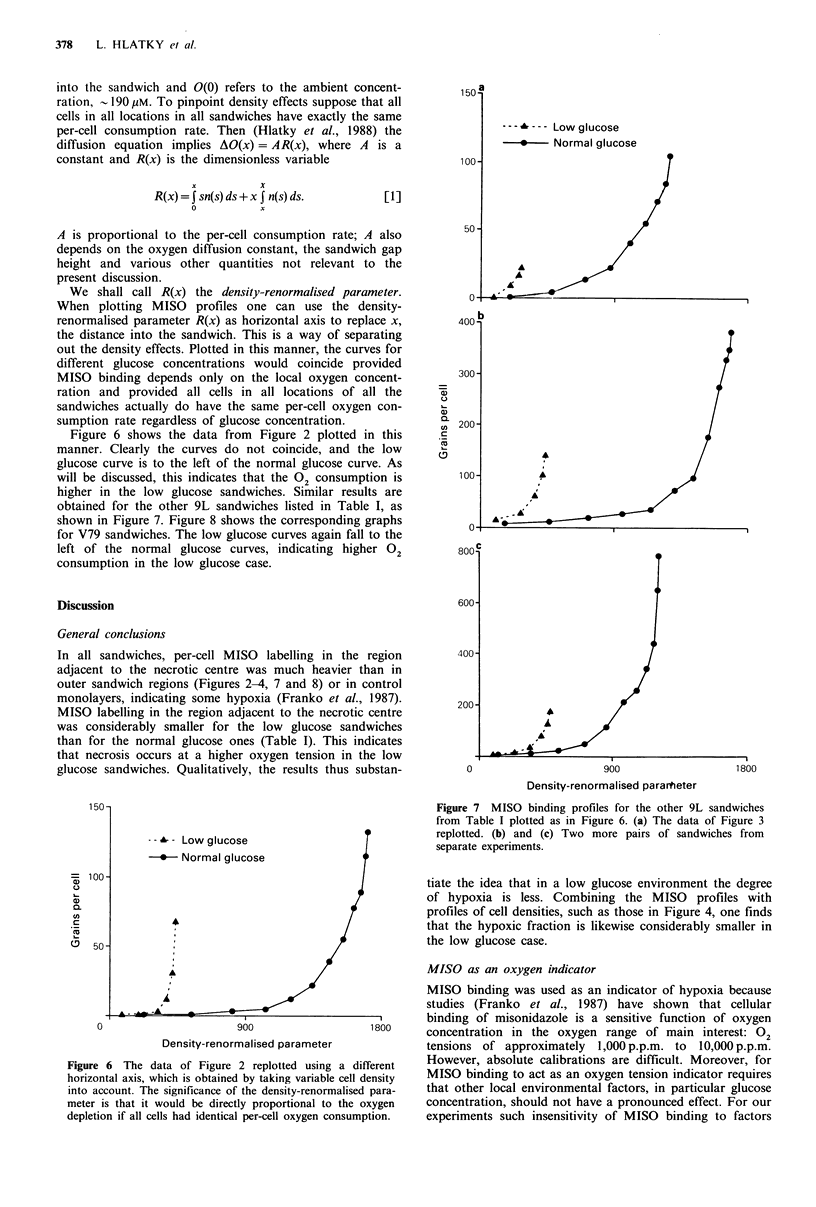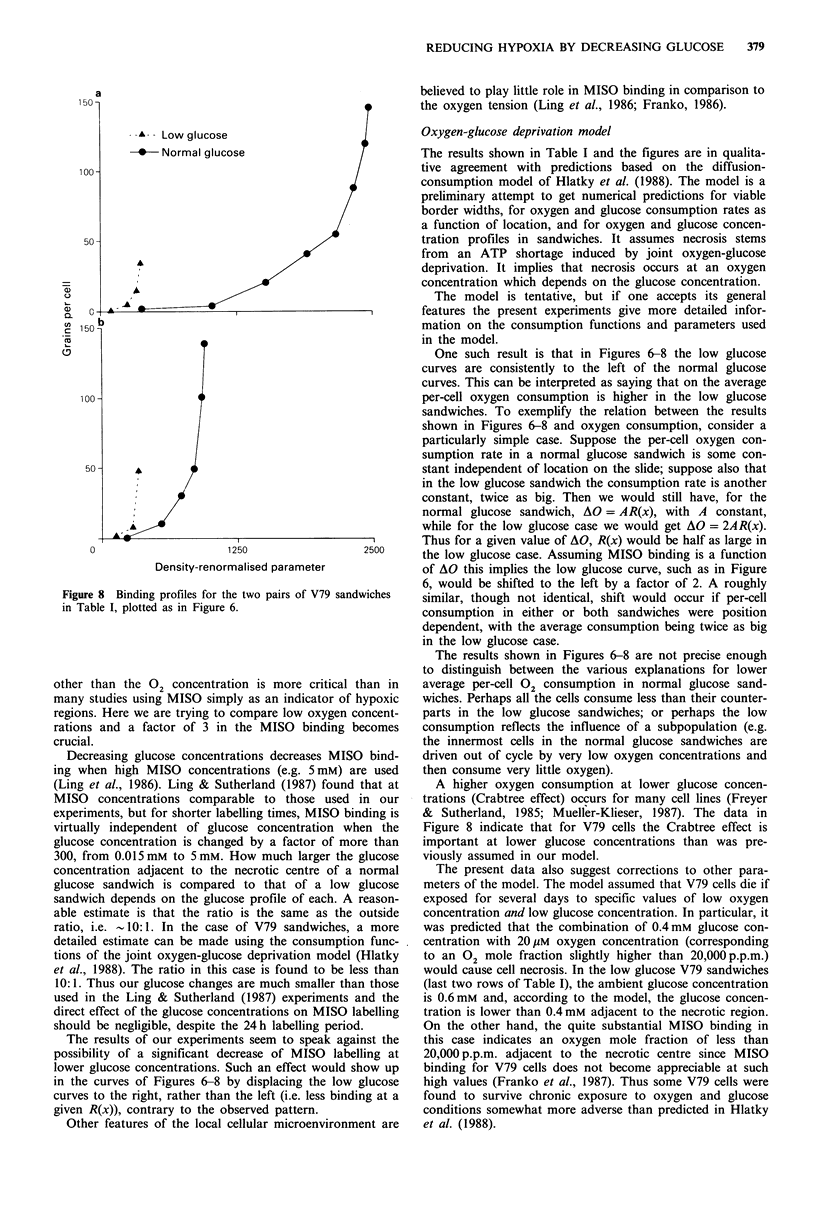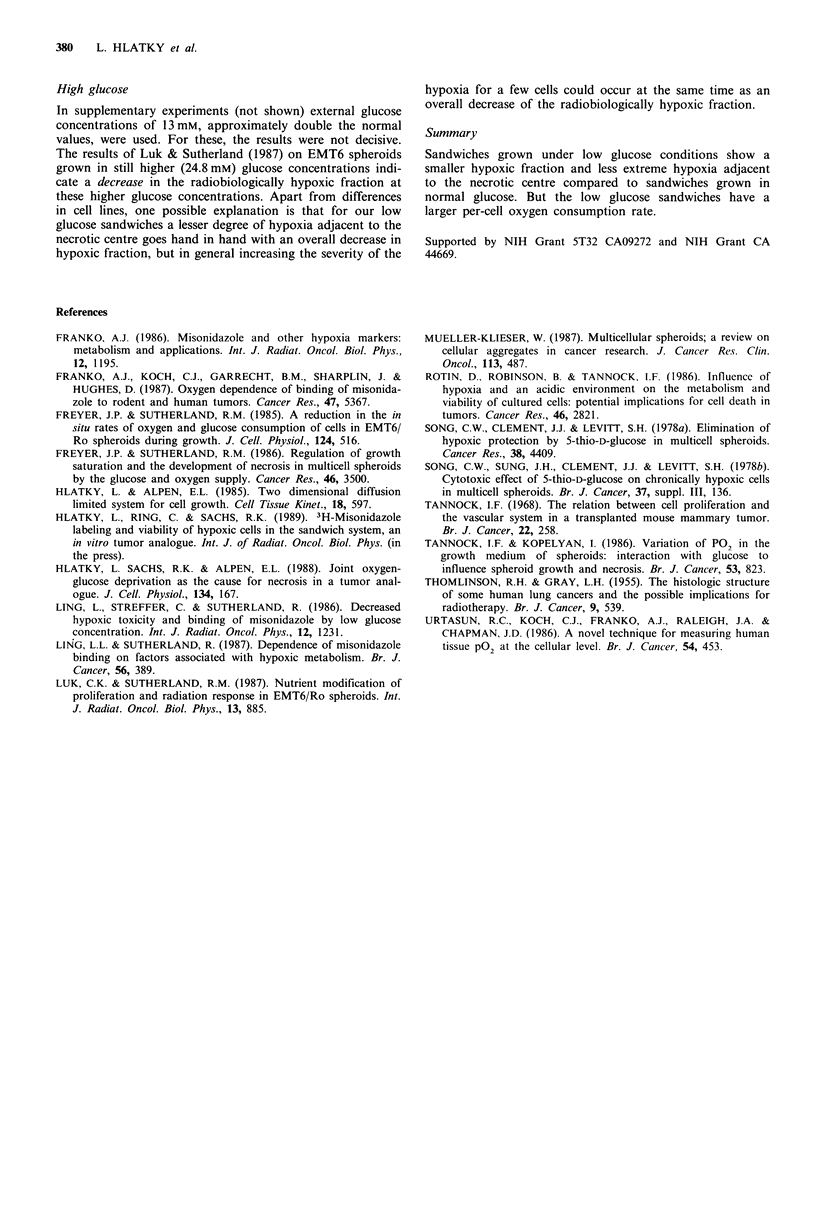# Reducing the hypoxic fraction of a tumour model by growth in low glucose.

**DOI:** 10.1038/bjc.1989.75

**Published:** 1989-03

**Authors:** L. Hlatky, R. K. Sachs, C. S. Ring

**Affiliations:** Cell and Molecular Biology Division, Lawrence Berkeley Laboratory, Boston, MA.

## Abstract

The question of whether growth under low glucose conditions leads to a reduced amount of cell hypoxia was investigated using an in vitro tumour analogue, the sandwich system. In this multicellular system, the interplay between diffusion and consumption of oxygen and nutrients results in spatial gradients of these environmental factors. Gradients in the environment lead to biological heterogeneity within the cell population. A necrotic centre, surrounded by a viable cell border, subsequently develops. Cells adjacent to the necrotic centre in sandwiches are hypoxic and are in an environment somewhat analogous to that of cells adjacent to necrotic regions in solid tumours. Using sandwiches of the 9L and V79 cell lines, the effects of growth under low glucose conditions on the degree of hypoxia in regions adjacent to the necrotic centre were investigated. Per-cell binding of 3H-misonidazole, assessed by autoradiography, was used as an indicator of oxygen deprivation. It was found that the extent of the hypoxic region and the severity of hypoxia were considerably reduced by growing sandwiches in a glucose concentration of 0.6 mM rather than 6.5 mM. This reduction was found in conjunction with a smaller viable border; it occurred despite the fact that the average per-cell oxygen consumption is higher in the low glucose sandwiches. The data are qualitatively consistent with a joint oxygen-glucose deprivation model for cell necrosis.


 © The Macmillan Press Ltd., 1989

### Reducing the hypoxic fraction of a tumour model by growth in low glucose

L. Hlatky[1,2], R.K. Sachs[3] & C.S. Ring[4]

[1]*Cell and Molecular Biology Division, Lawrence Berkeley Laboratory;* [2]*Joint Center for Radiation Therapy, Harvard Medical School, 50 Binney Street, Boston MA 02115;* [3] *Departments of Physics and Mathematics, University of California at Berkeley; and* [4]*Department of Pharmaceutical Chemistry, University of California at San Francisco, USA.*

**Summary** The question of whether growth under low glucose conditions leads to a reduced amount of cell hypoxia was investigated using an *in vitro* tumour analogue, the sandwich system. In this multicellular system, the interplay between diffusion and consumption of oxygen and nutrients results in spatial gradients of these environmental factors. Gradients in the environment lead to biological heterogeneity within the cell population. A necrotic centre, surrounded by a viable cell border, subsequently develops. Cells adjacent to the necrotic centre in sandwiches are hypoxic and are in an environment somewhat analogous to that of cells adjacent to necrotic regions in solid tumours. Using sandwiches of the 9L and V79 cell lines, the effects of growth under low glucose conditions on the degree of hypoxia in regions adjacent to the necrotic centre were investigated. Per-cell binding of $^3$H-misonidazole, assessed by autoradiography, was used as an indicator of oxygen deprivation. It was found that the extent of the hypoxic region and the severity of hypoxia were considerably reduced by growing sandwiches in a glucose concentration of 0.6 mM rather than 6.5 mM. This reduction was found in conjunction with a smaller viable border; it occurred despite the fact that the average per-cell oxygen consumption is higher in the low glucose sandwiches. The data are qualitatively consistent with a joint oxygen–glucose deprivation model for cell necrosis.

It is believed that for some tumours the radioresistance of hypoxic cells may be an important factor in limiting the success of radiotherapy. The difficulty comes in predicting which tumour types have significant numbers of radiobiologically hypoxic cells and in locating these cells. The presence of hypoxic cells near necrotic regions has long been suspected (Thomlinson & Gray, 1955; Tannock, 1968). But it is likely that not all hypoxic rgions border necrotic areas and it may be that not all necrotic areas are surrounded by hypoxia.

*In vitro* studies have shown strong evidence that other factors (e.g. glucose deprivation, pH), in addition to oxygen deprivation, are likely to play a significant role in necrosis formation in tumours (Freyer & Sutherland, 1986; Rotin *et al.*, 1986; Tannock & Kopelyan, 1986; Mueller-Klieser, 1987; Hlatky *et al.*, 1988*a*).

The realisation that more than oxygen deprivation is involved in triggering the onset of cell death and necrosis has implications for the amount of hypoxia one would expect to find surrounding necrotic regions. If oxygen deprivation alone causes necrosis then it follows that tumours would have severely hypoxic cells on the edge of all necrotic regions. But if other substances are involved, necrotic regions with little surrounding hypoxia could result in cases where factors other than the oxygen concentration are particularly unfavourable.

Related to the idea that there may not be significant areas of radiobiological hypoxia adjacent to a necrotic region if other factors (e.g. glucose deprivation) played a significant role in the cell necrosis, is the idea of intentionally decreasing the hypoxic fraction by starving hypoxic cells of glucose. Song *et al.* (1978*a*, *b*) argued that hypoxic cells should be particularly susceptible to glucose deprivation and found that the glucose analogue 5-thio-D-glucose was preferentially cytotoxic and radiosensitising for hypoxic cells.

In previous studies with tumour models grown under low glucose conditions, it was observed that spheroid viable rims (Freyer & Sutherland, 1986; Mueller-Klieser, 1987) and sandwich viable borders (Hlatky *et al.*, 1988) are narrower than when these tumour analogues are grown under normal glucose conditions. For the sandwiches this result was interpreted as being due to the cells dying as a result of a joint shortage of oxygen and glucose rather than due to oxygen deprivation alone (Hlatky *et al.*, 1988). It was predicted that cells in the low glucose sandwiches die at a higher oxygen tension and low glucose sandwiches should contain a smaller fraction of hypoxic cells than normal glucose sandwiches.

These predictions were tested by labelling sandwiches grown at two different glucose concentrations with $^3$H-misonidazole and assessing the amount and degree of hypoxia that results. Labelled misonidazole has been used as a marker for hypoxic cells. At oxygen tensions higher than those corresponding to radiobiological hypoxia, binding of MISO adducts is relatively small but at oxygen tensions comparable to those required for radiobiological hypoxia, much heavier binding is observed (Franko, 1986). Attempts are currently being made to assess radiobiological hypoxia in human tumours using $^3$H-MISO as a marker (Urtasun *et al.*, 1986).

### Materials and methods

### *Cell culture*

Two rodent cell lines, 9L and V79, were used for these studies. The 9L cell line originally came from an *N*-nitrosomethylurea induced gliosarcoma in a CD Fisher rat. The V79 cell line originated from Chinese hamster normal lung tissue. Monolayer and sandwich cultures were grown in Eagles MEM with Earle's salts (Gibco), supplemented with glutamine, 11% newborn calf serum (Gibco) and 4% fetal calf serum (Irvine Scientific); bicarbonate buffer was added. Medium of different glucose concentrations was obtained by using Eagle's MEM with Earle's salts but without glucose and adding powdered glucose. Cultures were incubated at 37°C in a humidified atmosphere of 5% $CO_2$ in air.

### *Sandwich system*

The sandwich system is an *in vitro* tumour analogue. Cells are grown in a diffusion limited environment. The result is a monolayer cell population that is heterogeneous with respect to nutrient and oxygen supply and therefore heterogeneous with respect to biological properties. The motivation for

Correspondence: L. Hlatky, Joint Center for Radiation Therapy, Harvard Medical School, 50 Binney Street, Boston MA 02115, USA.
Received 15 July 1988, and in revised form, 2 November 1988.

using such a system is that on a single microscope slide much of the diversity of microenvironments found in poorly vascularised tumours can be mimicked in an organised way. Any assay that can be conducted on monolayers can be used to analyse the sandwich cells, and cells from the different microenvironments can be separated. A brief description of the method is given here: for details see Hlatky & Alpin (1985). In the system, cells are grown sandwiched between two glass 1 × 3 inch microscope slides. The slides are separated by Teflon spacers of 150 $\mu$m. Cells are grown in monolayer on the bottom slide and medium completely fills the gap between the slides. The assemblage sits in a 3.5 × 3.5 inch Petri dish that acts as a medium reservoir. In order for the interior cells of the sandwich to be continuously supplied with nutrients, the nutrients must diffuse from the medium reservoir through the narrow gap between the slides (Figure 1). Due to competition between nutrient diffusion and nutrient consumption by the cells, gradients develop; in time the cells in the central region of the slide die forming the 'necrotic centre'. Live cells form a viable border, analogous to the viable rim in spheroids, within which gradients of morphology and ultastructure are observed.

In these studies, cells were initially seeded on the bottom slide at a density 0.5 × $10^6$ cells per slide. Following seeding, the slides were left uncovered for 24 h in order for the cells to resume exponential growth. At 24 h after seeding, top slides were added and the medium was changed. Medium with either low, 0.6 mM, or normal, 6.5 mM, glucose was added. Thereafter, the medium was not changed or supplemented. The oxygen and nutrient concentrations in the ambient medium outside the sandwich did not change appreciably during the course of an experiment.

### *$^3$H-misonidazole labelling*

Sandwich cultures and, as controls, uncovered monolayers were labelled with $^3$H-misonidazole (generously provided by Dr J.A. Raleigh and Dr A.J. Franko). The uptake of MISO as a function of distance into the sandwich was assessed using autoradiography. Sandwiches were labelled with the top slide in place. At the time of labelling, between 48 and 72 h after covering with the top slide, the sandwich is well-developed: it has a viable cell border, within which there is a gradient of local cell environments, and has a necrotic centre. Sandwiches and controls were labelled at a concentration of 60 $\mu$M (specific activity 584 $\mu$Ci mg$^{-1}$) for a 24 h period. A 24 h labelling period was chosen to allow adequate diffusion of the label into the interior regions of the sandwich. For example, a cell at a distance of 1 mm into the sandwich then experiences a time-averaged MISO concentration which is 89% of the concentration in the ambient medium outside the sandwich and a cell at a distance of 2 mm experiences a time-averaged concentration which is 78% of the outside concentration (Hlatky et al., 1989). The sandwiches were not removed from the incubator for labelling. Two ml of warmed medium containing the $^3$H-misonidazole was added to the culture dishes. The sandwiches were removed from the incubator only after the 24 h labelling. Following labelling, the radioactive medium was drawn off and the slides were rinsed twice with medium containing no MISO. The top slide was removed during this rinsing period, to allow maximum efficiency in rinsing out the unbound misonidazole. Following rinsing, medium without MISO was again added and the slides were incubated at 37°C in this medium for an additional 15 min. The slides were then rinsed three times in PBS, fixed in 3:1 ethanol:acetic acid, rinsed in 70% ethanol, air dried and dipped in NTB2 emulsion (Kodak).

**Figure 1** Cross-section of a sandwich. The cells are grown in monolayer attached to the bottom slide. $x$ is the distance from the medium reservoir to a cell; oxygen and nutrients diffuse inward in the positive $x$ direction and metabolites diffuse outward in the opposite direction.

The slides were exposed between 17 and 35 days depending on the experiment. Following development the slides were stained with haematoxylin.

Since in sandwiches cells are grown in a monolayer on the bottom slide, the scoring of sandwich slide autoradiographs is essentially the same as for control monolayer autoradiographs. Grains per cell are scored. Sandwich slides were scored for grains per cell as a function of distance into the sandwich. The viable border was subdivided into 0.2 mm strips for scoring purposes. The number of cells scored in a particular strip was typically between 20 and 40; in some cases, as many as 300 cells were scored to facilitate statistical analyses.

### Results

Sandwiches grown in various glucose concentrations were labelled with $^3$H-misonidazole and the MISO binding profiles were compared to determine the degree of hypoxia. In all cases, the outer sandwich cells, which are near the oxygen and nutrient source, exhibit very low MISO binding, analogous to that seen in the control monolayers, and the interior sandwich cells bordering the necrotic centre show significant amounts of bound MISO, indicative of hypoxia. Figure 2 shows the average number of grains per cell, as a function of the distance $x$ of the cells into the sandwich, for two 9L sandwiches. One sandwich is grown in low glucose, 0.6 mM; the other is in normal glucose, 6.5 mM.

Note that the width of the viable border for the low glucose sandwich is less than half that of the normal glucose sandwich, consistent with earlier observations (Hlatky et al.,

**Figure 2** Profiles of the number of MISO grains per cell in two 9L sandwiches, labelled 48 h after set-up. The horizontal axis gives the distance $x$ from the source of oxygen and nutrients to the observed cells. The necrotic centre was at 1.1 mm in the low glucose sandwich and at 2.4 mm in the normal glucose sandwich. Low glucose, 0.6 mM; normal glucose, 6.5 mM. For an exponentially cycling population the standard deviation $\sigma$ due to binding proportional to cell size is $\sigma \approx 0.2\ n$, where $n$ is the average number of grains counted per cell (Hlatky et al., 1989). In addition, Poisson fluctuations in the number of grains per cell counted produce an independent standard deviation $= \sqrt{n}$. These two sources of standard deviations are large enough to account for standard deviations of the size shown. The standard deviations reflect fluctuations in grains per cell counted within one experiment. There are of course other sources of error which are not reflected in these standard deviations.

1988) and consistent with the idea that the cells die due to a combination of low glucose and low oxygen. In the two sandwiches roughly the same MISO binding is observed at a given distance, but since the low glucose viable border is truncated relative to the normal glucose viable border the binding adjacent to the necrotic centre is smaller in the low glucose case.

The standard deviations are shown as error bars. There is evidence that much of the standard deviation in grain counts is due to variations in cell size, with larger cells binding more $^3$H-MISO (Hlatky *et al.*, 1989). There was a tendency to have more background grains in regions of higher MISO binding. No attempt to subtract background from grain counts was made.

Results similar to those shown in Figure 2 were obtained for other 9L sandwiches (e.g. Figure 3) and for V79 sandwiches (Figure 4). In all cases the maximum number of MISO grains per cell is considerably larger in the normal glucose sandwiches than in the low glucose sandwiches. Table I summarises data for the sandwiches shown in Figures 2–4 and for some further experiments. Note that the maximum per-cell binding in the low glucose sandwiches is 1/2 to 1/5 that of the normal glucose sandwiches. The maximum number of grains-per-cell, *N*, varies between experiments (rows of the table) due to differences in the age of sandwiches at labelling, length of exposure of the emulsion and sensitivity of the total grain counts to development conditions.

MISO binding profiles shown in Figures 2–4 reflect oxygen concentration profiles of the sandwiches. These oxygen concentration profiles in turn depend on cell density and on the per-cell oxygen consumption. Since cell densities are different under the different glucose conditions, direct comparison of MISO binding at a given distance is not the only informative way to compare the data. By taking into account the measured cell densities one can plot the data in a way which allows inferences to be drawn about the per-cell consumptions. Figure 5 shows the number $n(x)$ of cells per unit area at a distance $x$ into the sandwich for the sandwiches whose MISO binding is plotted in Figure 2. Cells were seeded uniformly but the adverse conditions at locations distant from the source of oxygen and nutrients lead to lower cell densities towards the interior of the sandwich. All sandwiches had density profiles of generally similar shape; for the smaller V79 cells the maximum number of cells per unit area was about twice that shown on this graph of 9L sandwiches.

The effect of variable cell density $n(x)$ and viable border width, $X$, on the oxygen concentration profile can be analysed by using the diffusion equation. We write the consumption induced oxygen depletion as $\Delta O(x) = O(0) - O(x)$ where $O(x)$ is the $O_2$ concentration at distance $x$

**Table I** Maximum number of grains per cell in low (0.6 mM) and normal (6.5 mM) glucose sandwiches. Each row represents a different experiment. Figure(s) in which the MISO labeling profile is shown. Line, cell line. L, maximum average number of grains per cell in a 0.2 mm wide strip for the low glucose sandwiches (in all cases this maximum occurred for the strip adjacent to the necrotic centre). N, the corresponding maximum for the normal glucose sandwiches. L/N, the ratio of the two maxima.

| *Figure* | *Line* | *L* | *N* | *L/N* (%) |
|---|---|---|---|---|
| 2,5,6 | 9L | 69 | 132 | 52 |
| 3,7a | 9L | 22 | 105 | 21 |
| 7b | 9L | 137 | 396 | 35 |
| 7c | 9L | 171 | 779 | 22 |
| 4,8a | V79 | 34 | 145 | 23 |
| 8b | V79 | 48 | 135 | 35 |

**Figure 3** Per-cell MISO labelling for two 9L sandwiches labelled 72 h after set-up. The glucose concentrations used were 0.6 mM and 6.5 mM. Viable border widths are smaller than for the sandwiches in Figure 2 labelled 48 h after set-up. The viable border width decreases in time as cells near the centre die off. Standard deviations for these sandwiches (not shown) were comparable to those in Figure 2.

**Figure 4** Per-cell MISO labelling for two V79 sandwiches labelled 48 h after set-up. Low glucose, 0.6 mM; normal glucose, 6.5 mM.

**Figure 5** Cell density profiles for the two 9L sandwiches shown in Figure 2.

into the sandwich and $O(0)$ refers to the ambient concentration, $\sim 190\,\mu M$. To pinpoint density effects suppose that all cells in all locations in all sandwiches have exactly the same per-cell consumption rate. Then (Hlatky et al., 1988) the diffusion equation implies $\Delta O(x) = AR(x)$, where $A$ is a constant and $R(x)$ is the dimensionless variable

$$R(x) = \int_0^x sn(s)\,ds + x \int_x^X n(s)\,ds. \qquad [1]$$

$A$ is proportional to the per-cell consumption rate; $A$ also depends on the oxygen diffusion constant, the sandwich gap height and various other quantities not relevant to the present discussion.

We shall call $R(x)$ the *density-renormalised parameter*. When plotting MISO profiles one can use the density-renormalised parameter $R(x)$ as horizontal axis to replace $x$, the distance into the sandwich. This is a way of separating out the density effects. Plotted in this manner, the curves for different glucose concentrations would coincide provided MISO binding depends only on the local oxygen concentration and provided all cells in all locations of all the sandwiches actually do have the same per-cell oxygen consumption rate regardless of glucose concentration.

Figure 6 shows the data from Figure 2 plotted in this manner. Clearly the curves do not coincide, and the low glucose curve is to the left of the normal glucose curve. As will be discussed, this indicates that the $O_2$ consumption is higher in the low glucose sandwiches. Similar results are obtained for the other 9L sandwiches listed in Table I, as shown in Figure 7. Figure 8 shows the corresponding graphs for V79 sandwiches. The low glucose curves again fall to the left of the normal glucose curves, indicating higher $O_2$ consumption in the low glucose case.

### Discussion

### *General conclusions*

In all sandwiches, per-cell MISO labelling in the region adjacent to the necrotic centre was much heavier than in outer sandwich regions (Figures 2–4, 7 and 8) or in control monolayers, indicating some hypoxia (Franko et al., 1987). MISO labelling in the region adjacent to the necrotic centre was considerably smaller for the low glucose sandwiches than for the normal glucose ones (Table I). This indicates that necrosis occurs at a higher oxygen tension in the low glucose sandwiches. Qualitatively, the results thus substantiate the idea that in a low glucose environment the degree of hypoxia is less. Combining the MISO profiles with profiles of cell densities, such as those in Figure 4, one finds that the hypoxic fraction is likewise considerably smaller in the low glucose case.

**Figure 6** The data of Figure 2 replotted using a different horizontal axis, which is obtained by taking variable cell density into account. The significance of the density-renormalised parameter is that it would be directly proportional to the oxygen depletion if all cells had identical per-cell oxygen consumption.

**Figure 7** MISO binding profiles for the other 9L sandwiches from Table I plotted as in Figure 6. (**a**) The data of Figure 3 replotted. (**b**) and (**c**) Two more pairs of sandwiches from separate experiments.

### *MISO as an oxygen indicator*

MISO binding was used as an indicator of hypoxia because studies (Franko et al., 1987) have shown that cellular binding of misonidazole is a sensitive function of oxygen concentration in the oxygen range of main interest: $O_2$ tensions of approximately 1,000 p.p.m. to 10,000 p.p.m. However, absolute calibrations are difficult. Moreover, for MISO binding to act as an oxygen tension indicator requires that other local environmental factors, in particular glucose concentration, should not have a pronounced effect. For our experiments such insensitivity of MISO binding to factors

**Figure 8** Binding profiles for the two pairs of V79 sandwiches in Table I, plotted as in Figure 6.

other than the $O_2$ concentration is more critical than in many studies using MISO simply as an indicator of hypoxic regions. Here we are trying to compare low oxygen concentrations and a factor of 3 in the MISO binding becomes crucial.

Decreasing glucose concentrations decreases MISO binding when high MISO concentrations (e.g. 5 mM) are used (Ling *et al.*, 1986). Ling & Sutherland (1987) found that at MISO concentrations comparable to those used in our experiments, but for shorter labelling times, MISO binding is virtually independent of glucose concentration when the glucose concentration is changed by a factor of more than 300, from 0.015 mM to 5 mM. How much larger the glucose concentration adjacent to the necrotic centre of a normal glucose sandwich is compared to that of a low glucose sandwich depends on the glucose profile of each. A reasonable estimate is that the ratio is the same as the outside ratio, i.e. ~10:1. In the case of V79 sandwiches, a more detailed estimate can be made using the consumption functions of the joint oxygen-glucose deprivation model (Hlatky *et al.*, 1988). The ratio in this case is found to be less than 10:1. Thus our glucose changes are much smaller than those used in the Ling & Sutherland (1987) experiments and the direct effect of the glucose concentrations on MISO labelling should be negligible, despite the 24 h labelling period.

The results of our experiments seem to speak against the possibility of a significant decrease of MISO labelling at lower glucose concentrations. Such an effect would show up in the curves of Figures 6–8 by displacing the low glucose curves to the right, rather than the left (i.e. less binding at a given $R(x)$), contrary to the observed pattern.

Other features of the local cellular microenvironment are believed to play little role in MISO binding in comparison to the oxygen tension (Ling *et al.*, 1986; Franko, 1986).

*Oxygen-glucose deprivation model*

The results shown in Table I and the figures are in qualitative agreement with predictions based on the diffusion-consumption model of Hlatky *et al.* (1988). The model is a preliminary attempt to get numerical predictions for viable border widths, for oxygen and glucose consumption rates as a function of location, and for oxygen and glucose concentration profiles in sandwiches. It assumes necrosis stems from an ATP shortage induced by joint oxygen-glucose deprivation. It implies that necrosis occurs at an oxygen concentration which depends on the glucose concentration.

The model is tentative, but if one accepts its general features the present experiments give more detailed information on the consumption functions and parameters used in the model.

One such result is that in Figures 6–8 the low glucose curves are consistently to the left of the normal glucose curves. This can be interpreted as saying that on the average per-cell oxygen consumption is higher in the low glucose sandwiches. To exemplify the relation between the results shown in Figures 6–8 and oxygen consumption, consider a particularly simple case. Suppose the per-cell oxygen consumption rate in a normal glucose sandwich is some constant independent of location on the slide; suppose also that in the low glucose sandwich the consumption rate is another constant, twice as big. Then we would still have, for the normal glucose sandwich, $\Delta O = AR(x)$, with $A$ constant, while for the low glucose case we would get $\Delta O = 2AR(x)$. Thus for a given value of $\Delta O$, $R(x)$ would be half as large in the low glucose case. Assuming MISO binding is a function of $\Delta O$ this implies the low glucose curve, such as in Figure 6, would be shifted to the left by a factor of 2. A roughly similar, though not identical, shift would occur if per-cell consumption in either or both sandwiches were position dependent, with the average consumption being twice as big in the low glucose case.

The results shown in Figures 6–8 are not precise enough to distinguish between the various explanations for lower average per-cell $O_2$ consumption in normal glucose sandwiches. Perhaps all the cells consume less than their counterparts in the low glucose sandwiches; or perhaps the low consumption reflects the influence of a subpopulation (e.g. the innermost cells in the normal glucose sandwiches are driven out of cycle by very low oxygen concentrations and then consume very little oxygen).

A higher oxygen consumption at lower glucose concentrations (Crabtree effect) occurs for many cell lines (Freyer & Sutherland, 1985; Mueller-Klieser, 1987). The data in Figure 8 indicate that for V79 cells the Crabtree effect is important at lower glucose concentrations than was previously assumed in our model.

The present data also suggest corrections to other parameters of the model. The model assumed that V79 cells die if exposed for several days to specific values of low oxygen concentration *and* low glucose concentration. In particular, it was predicted that the combination of 0.4 mM glucose concentration with 20 $\mu$M oxygen concentration (corresponding to an $O_2$ mole fraction slightly higher than 20,000 p.p.m.) would cause cell necrosis. In the low glucose V79 sandwiches (last two rows of Table I), the ambient glucose concentration is 0.6 mM and, according to the model, the glucose concentration is lower than 0.4 mM adjacent to the necrotic region. On the other hand, the quite substantial MISO binding in this case indicates an oxygen mole fraction of less than 20,000 p.p.m. adjacent to the necrotic centre since MISO binding for V79 cells does not become appreciable at such high values (Franko *et al.*, 1987). Thus some V79 cells were found to survive chronic exposure to oxygen and glucose conditions somewhat more adverse than predicted in Hlatky *et al.* (1988).

*High glucose*

In supplementary experiments (not shown) external glucose concentrations of 13 mM, approximately double the normal values, were used. For these, the results were not decisive. The results of Luk & Sutherland (1987) on EMT6 spheroids grown in still higher (24.8 mM) glucose concentrations indicate a *decrease* in the radiobiologically hypoxic fraction at these higher glucose concentrations. Apart from differences in cell lines, one possible explanation is that for our low glucose sandwiches a lesser degree of hypoxia adjacent to the necrotic centre goes hand in hand with an overall decrease in hypoxic fraction, but in general increasing the severity of the hypoxia for a few cells could occur at the same time as an overall decrease of the radiobiologically hypoxic fraction.

*Summary*

Sandwiches grown under low glucose conditions show a smaller hypoxic fraction and less extreme hypoxia adjacent to the necrotic centre compared to sandwiches grown in normal glucose. But the low glucose sandwiches have a larger per-cell oxygen consumption rate.

Supported by NIH Grant 5T32 CA09272 and NIH Grant CA 44669.

**References**

FRANKO, A.J. (1986). Misonidazole and other hypoxia markers: metabolism and applications. *Int. J. Radiat. Oncol. Biol. Phys.*, **12**, 1195.

FRANKO, A.J., KOCH, C.J., GARRECHT, B.M., SHARPLIN, J. & HUGHES, D. (1987). Oxygen dependence of binding of misonidazole to rodent and human tumors. *Cancer Res.*, **47**, 5367.

FREYER, J.P. & SUTHERLAND, R.M. (1985). A reduction in the *in situ* rates of oxygen and glucose consumption of cells in EMT6/Ro spheroids during growth. *J. Cell. Physiol.*, **124**, 516.

FREYER, J.P. & SUTHERLAND, R.M. (1986). Regulation of growth saturation and the development of necrosis in multicell spheroids by the glucose and oxygen supply. *Cancer Res.*, **46**, 3500.

HLATKY, L. & ALPEN, E.L. (1985). Two dimensional diffusion limited system for cell growth. *Cell Tissue Kinet.*, **18**, 597.

HLATKY, L., RING, C. & SACHS, R.K. (1989). $^{3}$H-Misonidazole labeling and viability of hypoxic cells in the sandwich system, an *in vitro* tumor analogue. *Int. J. of Radiat. Oncol. Biol. Phys.* (in the press).

HLATKY, L. SACHS, R.K. & ALPEN, E.L. (1988). Joint oxygen-glucose deprivation as the cause for necrosis in a tumor analogue. *J. Cell. Physiol.*, **134**, 167.

LING, L., STREFFER, C. & SUTHERLAND, R. (1986). Decreased hypoxic toxicity and binding of misonidazole by low glucose concentration. *Int. J. Radiat. Oncol. Phys.*, **12**, 1231.

LING, L.L. & SUTHERLAND, R. (1987). Dependence of misonidazole binding on factors associated with hypoxic metabolism. *Br. J. Cancer*, **56**, 389.

LUK, C.K. & SUTHERLAND, R.M. (1987). Nutrient modification of proliferation and radiation response in EMT6/Ro spheroids. *Int. J. Radiat. Oncol. Biol. Phys.*, **13**, 885.

MUELLER-KLIESER, W. (1987). Multicellular spheroids; a review on cellular aggregates in cancer research. *J. Cancer Res. Clin. Oncol.*, **113**, 487.

ROTIN, D., ROBINSON, B. & TANNOCK, I.F. (1986). Influence of hypoxia and an acidic environment on the metabolism and viability of cultured cells: potential implications for cell death in tumors. *Cancer Res.*, **46**, 2821.

SONG, C.W., CLEMENT, J.J. & LEVITT, S.H. (1978*a*). Elimination of hypoxic protection by 5-thio-D-glucose in multicell spheroids. *Cancer Res.*, **38**, 4409.

SONG, C.W., SUNG, J.H., CLEMENT, J.J. & LEVITT, S.H. (1978*b*). Cytotoxic effect of 5-thio-D-glucose on chronically hypoxic cells in multicell spheroids. *Br. J. Cancer*, **37**, suppl. III, 136.

TANNOCK, I.F. (1968). The relation between cell proliferation and the vascular system in a transplanted mouse mammary tumor. *Br. J. Cancer*, **22**, 258.

TANNOCK, I.F. & KOPELYAN, I. (1986). Variation of $PO_2$ in the growth medium of spheroids: interaction with glucose to influence spheroid growth and necrosis. *Br. J. Cancer*, **53**, 823.

THOMLINSON, R.H. & GRAY, L.H. (1955). The histologic structure of some human lung cancers and the possible implications for radiotherapy. *Br. J. Cancer*, **9**, 539.

URTASUN, R.C., KOCH, C.J., FRANKO, A.J., RALEIGH, J.A. & CHAPMAN, J.D. (1986). A novel technique for measuring human tissue $pO_2$ at the cellular level. *Br. J. Cancer*, **54**, 453.